# Effective population size mediates the impact of pollination services on pollen limitation

**DOI:** 10.1098/rspb.2023.1519

**Published:** 2024-01-10

**Authors:** Anita Cisternas-Fuentes, Matthew H. Koski

**Affiliations:** ^1^ Department of Biological Sciences, Clemson University, Clemson, SC, USA; ^2^ Departamento de botánica, Facultad de Ciencias Naturales y Oceanográficas, Universidad de Concepción, Concepción, Chile

**Keywords:** clonality, genetic diversity, plant reproduction, pollination, pollen limitation, self-incompatibility

## Abstract

Inadequate pollen receipt limits flowering plant reproduction worldwide. Ecological causes of pollen limitation (PL), like pollinator scarcity and low plant abundance, have been a primary research focus. The genetic diversity of plant populations could impact both quantity and quality components of PL in concert with ecological factors, yet empirical examples are lacking. We evaluated joint effects of ecological factors (flower abundance, pollinator visitation) and genetic effective population size (*N_E_*) on PL across 13 populations of the common herb *Argentina anserina*. We used a histological approach with 5504 styles from 1137 flowers to separate quantity and quality components of PL, and link these to reproductive output. *N_E_* and pollinator visitation interacted to shape PL, but *N_E_* had stronger direct effects. Effectively smaller populations experienced stronger quantity PL, and controlled crosses in a pollinator-free environment revealed that stigmatic pollen quantity was an intrinsic population-level attribute that increased with *N_E_*. Pollinator visitation enhanced pollen quality, but only in effectively larger populations. Quantity and quality PL negatively impacted fruit and seed set, respectively. Results highlight that PL is dictated by plant population genetic diversity in addition to commonly evaluated ecological factors. Efforts to support pollinators will more strongly enhance plant reproduction in genetically diverse populations.

## 1. Introduction

Animal-mediated pollination is crucial for the sexual reproduction of almost 90% of flowering plants [1], but factors such as pollinator declines and habitat fragmentation can have negative consequences for plant–pollinator mutualisms [2]. Pollen limitation (PL), a reduction in seed production due to inadequate pollen receipt, is pervasive among flowering plants and contributes to selection on floral traits [3], mating system evolution [4], and species range dynamics [5]. The ecological causes of PL, and intrinsic plant attributes that shape variation in PL (e.g. reliance on pollinators) have been primary areas of research over the past decades [6,7]. The genetic diversity of plant populations is predicted to impact PL [8] but has been largely overlooked empirically. While several studies have demonstrated reductions in plant reproductive output from populations with smaller census size [9–12], mechanisms underlying this relationship are often unexplored (however see [13]) and census size does not necessarily reflect genetic effective population size [14]. As land use change and climate change pose threats to plant population genetic diversity [15,16], an understanding of its impact on PL of plant reproduction is imperative.

PL results from the receipt of fewer pollen grains than available ovules (pollen quantity limitation), or the receipt of low-quality pollen that fails to fertilize ovules (pollen quality limitation) [6]. Both components of PL are well known to be impacted by ecological factors that dictate pollen availability within a population and pollen transfer between flowers. For instance, few reproductive plants within a population (e.g. [17]), low pollinator abundance (e.g. [18]), inefficient pollinators (e.g. [19]) and the deposition of heterospecific pollen (e.g. [20]) can exacerbate both pollen quantity and quality limitation. Although we lack strong empirical evidence, genetic attributes of plant populations have the potential to shape variation in PL either alone or in concert with ecological factors. The genetic effective population size of plants may shape variation in PL in multiple ways. First, in species with the capacity for clonality (e.g. approx. 80% of angiosperms in Central Europe [21]), populations that are strongly clonal can evolve to invest less in sexual reproduction [22–24]. Such divestment in sex could come with a reduction in pollen production [25], and thus exacerbate pollen quantity limitation. Second, the genetic diversity of the stigmatic pollen load, a reflection of population genetic diversity at large, has the potential to shape the likelihood of fertilization. Genetically diverse stigmatic pollen loads should increase pollen competition, increasing overall quality of the pollen tubes reaching ovules [8]. Finally, in self-incompatible (SI) taxa (approx. 39% of angiosperm species globally [26]), the receipt of pollen with shared self-incompatibility alleles (S-alleles) prevents fertilization. Because S-allele diversity positively covaries with genetic population size [27], the likelihood of receiving high quality compatible pollen should also increase with genetic effective population size.

PL is commonly measured by comparing seed production from outcross-supplemented flowers with those left to open-pollination, which measures the combined impacts of pollen quantity and quality limitation on female reproductive success [28]. An approach that relates the number of pollen tubes that successfully reach ovules (a prezygotic estimate of fertilization success) to the number of pollen grains deposited on stigmas parses the contributions of both the pollen quantity and quality to overall PL [29]. Specifically, fewer stigmatic pollen grains indicates stronger pollen quantity limitation while a higher proportion of stigmatic pollen grains that successfully grow tubes and reach the ovule indicates higher quality of the stigmatic pollen load. Using the pollen grain-to-tube relationship to measure PL reduces variation in PL caused by resource limitation and allocation which can be strong when estimating PL by comparing seed production between outcrossed and open-pollinated flowers [7,29]. Combining prezygotic estimates of pollen quantity and quality limitation with post-zygotic metrics of reproductive success like fruit and seed set can be a powerful way to estimate the degree to which pollen quantity and quality each impact female reproductive output. The relationship between stigmatic pollen load and seed production is commonly assessed in the literature [13,30–34]; however, connecting both quantity and quality components of PL with seed production is rarely done.

**Figure 1.**
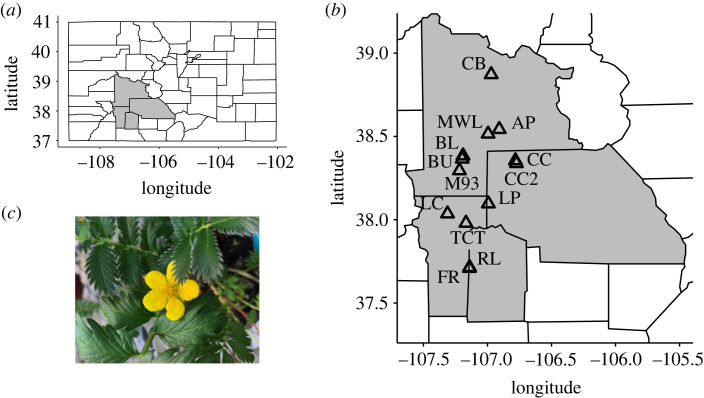
(*a*) A map of Colorado, USA, with four counties in grey depicting the focal region of *Argentina anserina* populations used in this study, (*b*) location of 13 focal populations and (*c*) an image of cosexual *A. anserina* flower.

Here, we combine genomic estimates of effective population size, ecological data on pollinator visitation and flower density, and histological data on pollen–pistil interactions to demonstrate how population genetic and ecological factors together shape components of PL and downstream reproductive output across 13 populations of *Argentina anserina*, a vegetatively clonal herb with a gametophytic self-incompatibility mechanism (figure 1). We tested the following predictions. (1) Pollen quantity and quality limitation will be exacerbated in populations that experience low pollinator visitation rates and those with small effective population size. (2) Pollinator visitation and effective population size will interact to shape pollen quality limitation. Specifically, high pollinator visitation rates should more effectively alleviate PL in genetically diverse populations (higher effective population size) relative to less diverse populations. (3) Both pollinator visitation and plant effective population size will mediate female reproductive output via their impacts on pollen–pistil interactions.

## 2. Methods

### (a) Study system and populations

*Argentina anserina* (L.) Rydb. (Rosaceae) is a perennial herb common to temperate climates of both the Northern and Southern Hemispheres [35]. Reproduction occurs both sexually and asexually via vegetative propagation. The flowers are cosexual, largely SI [36] and predominantly pollinated by small solitary bees and flies [37]. Asexual reproduction occurs through stolons [38] and vegetative clonal vigour varied among populations in a common greenhouse environment [39]. Flowers consist of many pistils, each with a single style and ovule. Flowers are receptive for 2 days. On the first day of floral anthesis anthers dehisce, and stigmas are receptive [36]. Fruits are an aggregate cluster of dry single-seeded achenes that fully develop about two weeks after pollination.

In Colorado, *Argentina anserina* inhabits pond and river edges, and wet meadows. The flowering season is mid-June to the end of July. In the San Juan Mountains of southwestern Colorado, populations occur between 1900 and 3500 metres above sea level (m.a.s.l.). In this study, we collected data in 13 populations spanning the species' elevational range in the focal region (figure 1). The lowest elevation populations were in the Gunnison River Valley (near 2300 m.a.s.l.) and the highest elevation populations were at edges of high elevation kettle ponds or rivers in the San Juan Mountains (3400 m.a.s.l.). Any given population was separated from others by more than 500 m of land unoccupied by *A. anserina*.

### (b) Style processing and scoring

To quantify components of pollen quality and quality limitation in *A. anserina* populations, we assessed prezygotic stages of fertilization success from open-pollinated flowers collected in the field [29,36,40–44]. We collected styles from senesced flowers in 13 populations of *A. anserina* during peak flowering in July of 2019, 2020 and 2021. We collected flowers at least 1 m apart to reduce the likelihood of re-sampling ramets of the same genet. We placed styles in vials with 70% ethanol (EtOH), and transported them to the laboratory at Clemson University. Across populations and years, we processed and scored 5504 styles from 1137 flowers (electronic supplementary material, table S1). In each population, we sampled between 39 to 145 flowers (mean = 87.46). In 2019, we collected and processed 1790 styles from 366 flowers across eight populations, in 2020 we collected and processed 1545 styles from 322 flowers across 11 populations, and in 2021 we collected and processed 2169 styles from 449 flowers across 10 populations.

We stained field-collected styles using decolorized aniline blue (DAB) to score pollen deposition, germination, and tube growth within styles. Before staining, we washed styles with deionized water, softened them using a 0.5 N solution of potassium hydroxide (KOH), and placed in a hot water bath at 65°C for 20 min. We then removed KOH, added DAB, and placed tubes again in a hot water bath at 65°C for 20 min. Five styles per flower were mounted on a microscope slide, gently squashed under a coverslip and sealed with clear nail polish.

We observed stained styles under a Zeiss AxioLab 5 microscope (Jena, Germany) using UV fluorescence to visualize pollen tubes. We collected data on stigmatic pollen deposition and the number of pollen tubes at the base of the style. Pollen deposition was scored by counting the number of pollen grains adhered to the stigmatic surface. Heterospecific pollen grains were very rare (present on less than 1% of styles scored) and were excluded from the number of grains deposited on stigmas. For each flower, we averaged the number of stigmatic pollen grains, and pollen tube number across the 5 styles scored. Previous work using a subset of the samples presented here showed that results from analyses of style-level and flower-level average data were similar [36].

### (c) Effective population size

Within- and between-population metrics of genetic diversity for the 13 focal populations were evaluated in Cisternas-Fuentes & Koski [39] using ddRadSeq. Briefly, we extracted genomic DNA from 6 to 8 individuals per population, at least 2 m distance from one another. We used *PstI* and *MseI* restriction enzymes*,* and samples were identified by a unique 11 bp barcode. The library was generated and sequenced by using llumina HiSeq by Floragenex, Inc. (Portland, Oregon, USA).

We used STACKS v. 2.53 to process the ddRadSeq and using the filtering parameter provided in Cisternas-Fuentes & Koski [39], we obtained a matrix of 5218 SNPs that were variable across populations and this matrix was used to obtain within population genetic diversity estimates. For the current study, we were particularly interested in the estimates of genetic effective population size (*N_E_*) as it may impact metrics of PL. Highly clonal populations should have smaller *N_E_* which could reduce pollen quantity, and populations with smaller *N_E_* should have lower pollen quality due to few S-alleles and lower pollen competition. We used the heterozygote excess model to estimate *N_E_* (*N_E_*Estimator v. 2.1 [45]). This approach estimates the effective number of breeding individuals in a population assuming fewer breeders when heterozygosity is higher than expected under Hardy–Weinberg equilibrium. Higher heterozygote excess is an indicator of reduced effective population size, and in clonal species, higher clonality [46,47]. One population was estimated to have an effective population size of ‘infinity' which can be interpreted as a population in which a limited number of parents does not cause a reduction in heterozygosity (i.e. the population is large) [45]. We bound this population to the largest of all estimated effective population sizes (*N_E_* = 15) as a conservative estimate.

### (d) Pollinator visitation

We recorded pollinator visitation to flowers of *A. anserina* in each population in summer 2020 and 2021 to quantify variation in visitation rate among populations. During peak pollinator activity (10.00–16.00), we selected 1 m × 1 m patches of flowering plants in each population, counted the number of open flowers, and recorded visitation for 5 min. Every pollinator that contacted a flower of *A. anserina* in the patch was recorded. Each visit to a separate flower by the same individual pollinator was scored as a separate visit. We categorized pollinator identity broadly as bee or fly, both of which can effectively deposit and export pollen in *A. anserina* [37]. We observed a total of 2075 flowers across 171 patches with an average of 13.16 patches (range per population 6–18; s.e. = 1.22) and 159.61 (s.e. = 38.27) flowers per population. In total, we recorded visitation for 860 min (mean of 66.15 min per population; s.e. = 5.89). At the patch level, we calculated visitation rate by bees, flies, and both pollinator classes combined as visits per flower per 5 min observation period.

### (e) Flower density

Because flower density within a population may impact PL [6], we explored its utility as a covariate in models explaining variation in PL among populations. To measure flower density, we ran two 50 m transects through each population during peak flowering in 2021, and every 10 m we counted flowers within a 1 m × 1 m plot (see [39]). Average plot level flower number was used as a metric of population-level flower density.

### (f) Fruit and seed set

We recorded fruit and seed set in summer 2022 near the end of the flowering season in each population (mid- to late July). To estimate fruit set, we identified 15 plants per population, each at least 2 m apart. We counted the total number of senesced flowers produced per plant, and the total number of fruits produced. We collected 1–3 fruits from each plant that successfully produced fruit. Fruits were allowed to dry for one month and then we counted seeds per fruit. Seed number was divided by the average ovule number in a given population to estimate seed set. Average ovule number per flower in each population was determined by collecting fresh flowers from the field in 2021, storing them in 70% EtOH, and counting ovules under a dissecting microscope [36]. The number of flowers scored per population ranged from 6 to 42 (mean = 12.4 per population). In one population (M93) flooding during summer 2022 resulted in the inability to collect fruit and seed set data. For this population, we used reproductive data from 2021 collected for a different project to estimate fruit and seed set. Specifically, individual flowers were tagged throughout the population during anthesis. They were then revisited at the end of the season and collected to score fruit set and seed number. Thus, fruit and seed set in M93 were estimated at the flower level rather than the plant level. We explored statistical models with and without reproductive data from this population, and its inclusion had no qualitative effect on results, thus this population was retained in final analyses.

### (g) Linking field pollen quantity and greenhouse pollen quantity

After finding a strong positive association between *N_E_* and pollen quantity (stigmatic pollen grain number) in the field (see Results), we investigated whether our metric of pollen quantity, stigmatic pollen load, is a genetic attribute of populations, and whether it is associated with *N_E_*. Plants from all focal populations studied in the field were collected between 2019 and 2021 and retained as stock populations in the greenhouse at Clemson University. As part of other studies, we conducted within-population controlled crosses in a pollinator-free greenhouse (e.g. [36]), and counted pollen deposited per stigma in the same manner as described above for field-collected stigmas. All crosses were conducted using the same size and brand of horsehair brush after anthers from a single flower from the pollen donor were allowed to dry for 24 h. Because pollen deposition was scored using the same pollination mode in every cross, the primary source of variation in stigmatic pollen load size among populations should be driven primarily by the number of pollen grains produced by flowers in a given population. We combined data from 1730 styles from 360 crosses across eight populations (mean = 45 per population) to estimate average pollen deposition per population in a common environment.

### (h) Analyses

All analyses described below were conducted in R v. 4.2.2.

#### (i) Pollen quality and quantity limitation

We estimated metrics of pollen quantity and quality limitation for each population by averaging flower-level pollen deposition and pollen tube number at the base of pistils. Pollen quantity was defined as the average number of grains deposited per stigma across all flowers collected in a given population [36]. To estimate a pre-fertilization metric of pollen quality, we used the piece-wise regression approach of Alonso *et al*. [29] within each population [36,41–44]. For each population, we modelled the number of pollen tubes at the base of styles as a function of pollen grains adhered to stigmas using a segmented regression (segmented package [48]). Parameters (*b1*, *k*, *c* and *b2*) were estimated using 1000 bootstraps. The first slope of pollen tubes on pollen grains, *b1*, estimates pollen quality. A higher *b1* indicates higher pollen quality because more pollen tubes reach the ovary per pollen grain deposited [29]. Values *c* and *k* represent the number of pollen grains and tubes, respectively, where the initial *b1* slope ceases to explain the relationship between tubes and grains (breakpoint). To standardize estimates of *b1* across populations, we forced the *y*-intercept to zero. In one population (M93), a linear model of pollen tubes on pollen grains was better supported than a piece-wise model because a breakpoint was not reached. For that population, we used the slope of the linear regression of tubes on grains as a metric of pollen quality.

For our estimation of population-level pollen quantity and quality limitation, we combined all flowers collected across years in each population (1–3 years per population; electronic supplementary material, table S1). Prior to grouping samples across years within populations, we assessed interannual variation in pollen quantity and quality at the population level. We ran the piece-wise regressions described above within each year and population to estimate the *b1* slope, and averaged pollen deposition per style at the population and year levels. We found that for populations with at least 2 years of data (11 of 13 focal populations), pollen quality and quantity estimates were strongly correlated across years (pollen quality: *r =* 0.824*, p* = 0.002; pollen quantity*: r* = 0.64*, p* = 0.035) (electronic supplementary material, table S2)*.* This supported that estimates of pollen quantity and quality limitation were repeatable across years within populations. Moreover, Alonso *et al*. [29] recommend 150–200 samples for the construction of the piece-wise regression. Combining data across years increased sample size within populations providing more robust estimates of pollen quantity and quality limitation.

#### (ii) Pollinator visitation

To determine whether pollinator visitation rates varied among populations, we modelled pollinator visitation rate (visits per flower) from each 5 min observation period (*N* = 172) as a function of population identity and the following covariates: time of day and its squared term to account for a curvilinear relationship between visitation and time of day, temperature, cloud cover, wind and year of observation. Cloud cover was categorized as full sun, partly cloudy or overcast. Wind was scored as a binary factor—windy (greater than 19 km h^−1^) or not (less than 19 km h^−1^). Visitation rate was right-skewed, so we modelled it using a Poisson distribution with a generalized linear model (R, ‘glm') which generated normally distributed residuals. From this model, we generated estimated marginal means of population-level visitation rates using *emmeans*. Bee and fly visitation rates were combined because a preliminary model did not support that populations differed in the relative visitation by bees and flies (Population × Pollinator Class, *X*^2^ = 11.88, *p* = 0.45) and previous work showed that both are effective pollinators [37]. Furthermore, a preliminary model with a dataset of populations with visitation recorded in two separate years (*N* = 8) revealed that visitation rates did not differ between years at the population level (Population × Year *X*^2^ = 8.11, *p* = 0.32). Together, these preliminary models justified grouping visitation rates across pollinator classes and years.

#### (iii) Linking pollination and effective population size to reproduction through pollen limitation

We used structural equation modelling (SEM) to determine (1) the effects of pollinator visitation, effective population size, and their interaction on both the quantity and quality components of PL and (2) the effects of each component of PL on fruit set and seed set. Specifically, we constructed a model with average population pollen quantity (average pollen deposition per stigma) and pollen quality (*b1* slope) each modelled as a function of pollinator visitation rate, effective population size, and their interaction term, accounting for the correlation between predictor variables. In the SEM, we additionally modelled fruit set and seed set as functions of pollen quantity and pollen quality. Together this model allowed us to determine the direct and interactive effects of each predictor on PL and reproductive output. We included an interaction term between pollinator visitation rate and *N_E_* [49] because we specifically predicted that increased pollinator visitation would enhance pollen quality only in populations with higher *N_E_*. The SEM was constructed and tested using the *lavaan* package in R [50]. A model that included flower density in each population as a predictor of pollen quantity and quality yielded a poorer model fit than the model without flower density (AIC with flower density = 28.72; AIC without flower density = 25.45), and flower density had no effect on pollen quantity (*p* = 0.40) or quality (*p* = 0.82) (electronic supplementary material, table S3). Thus, we present results of the simpler SEM without flower density (electronic supplementary material, table S4).

#### (iv) Linking field pollen quantity and greenhouse pollen quantity

We correlated average population-level pollen quantity in the field with average population-level pollen quantity from controlled crosses in the greenhouse, and with N_E_ using Pearson product-moment correlation (R ‘cor.test'). If among-population variation in stigmatic pollen load following controlled crosses is driven by differences in pistils per flower instead of pollen production, then populations with fewer pistils per flower should have higher pollen loads. Therefore, we correlated average stigmas per flower with average stigmatic pollen load size at the population level. Pistil number per flower was unassociated with pollen deposition in the field (*r* = 0.025, *p* = 0.94, d.f. = 11) and in the greenhouse (*r* = −0.26, *p* = 0.52, d.f. = 6). Therefore, we did not account for stigma number when assessing relationships between pollen deposition in the greenhouse, field, and *N_E_*.

## 3. Results

### (a) Variation in pollinator visitation and effective population size

Pollinator visitation rate varied significantly among populations (*X*^2^ = 26.54, *p* = 0.009), and was influenced by time of day (time *X*^2^ = 6.38 *p* = 0.012, time^2^ *X*^2^ = 6.08, *p* = 0.014) and year (*X*^2^ = 25.65, *p* < 0.0001) but not temperature, wind or cloud cover (all *p* > 0.49). Accounting for effects of time of day and year, the estimated marginal mean of pollinator visitation rate varied over 10-fold among populations (0.113–1.23 visits/flower/5 min).

Genomic estimates of effective population sizes were small, ranging between 1.1 and 15. Effective population size and pollinator visitation rate were positively correlated (*r* = 0.715, *p* < 0.01).

### (b) Variation in pollen limitation, fruit set and seed set

**Figure 2.**
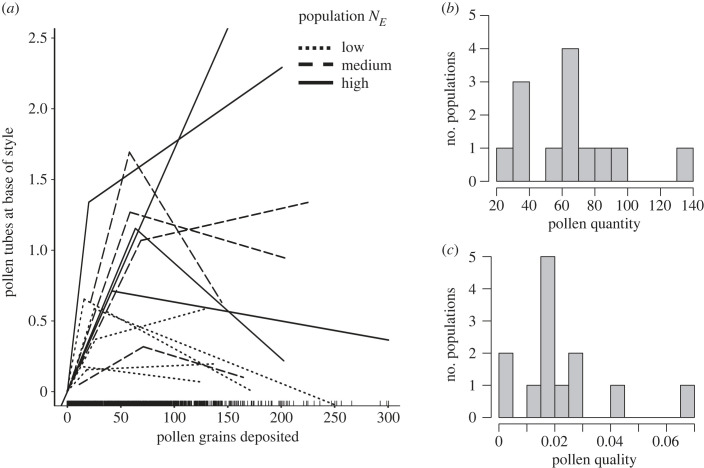
(*a*) Segmented relationships between pollen deposition per pistil and number of pollen tubes at the base of the style for 13 populations of *Argentina anserina*. Variation in the number of pollen tubes from all but one population (M93) was explained by a segmented relationship with two distinct slopes. Pollen quantity at the population level was estimated by the average number of grains deposited per stigma, and pollen quality was estimated by the initial slope of the segmented regression of pollen tubes on pollen grains deposited on stigmas, or simply the linear regression for M93. Line type depicts populations by effective population size: low (*N_E_* < 4); medium (4 < *N_E_* < 5.3); and high (*N_E_* > 5.8). The frequency distributions of (*b*) pollen quantity and (*c*) pollen quality among populations.

Across populations, the average number of pollen grains deposited per stigma (pollen quantity) varied over fivefold, from 23.39 to 130.79 (figure 2*b*). The *b1* slope of pollen tubes at the base of stigmas on the number of grains deposited (pollen quality) varied over 20-fold among populations, from 0.003 to 0.67 (figure 2*a*,*c*). Pollen quantity and quality were not correlated (*r* = −0.004, *p* = 0.99). The failure to receive any pollen at the flower level was exceedingly rare (1.6% of all 1137 flowers). Across populations the frequency of flowers that did not receive pollen ranged from 0% to 5.7%. Failure to have any fully developed pollen tubes at the flower level was more common (38.8% of all flowers) and ranged from 4.8% to 79.5% across populations. Fruit set ranged from 0 to 0.76 across populations while seed set ranged from 0 to 0.81. Fruit set and seed set were positively correlated, but not significantly so (*r* = 0.46, *p* = 0.12).

### (c) Linking pollination and effective population size to reproduction through pollen limitation

**Figure 3.**
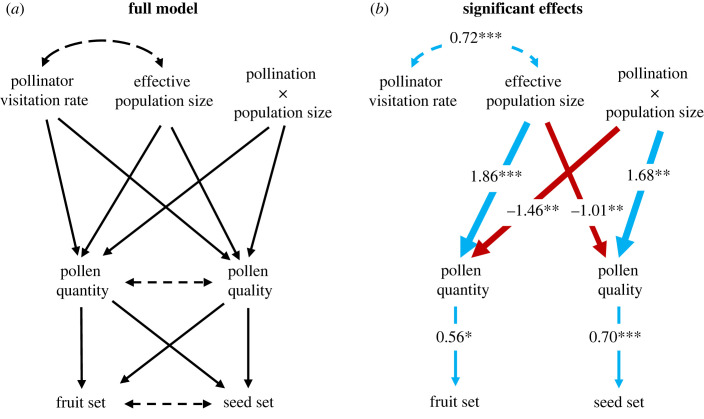
(*a*) Path diagram of the full structural equation model linking pollinator visitation rate, plant effective population size, and their interaction to seed and fruit set via both quantitative and qualitative metrics of pollen limitation. Solid lines depict direct effects and dashed lines depict correlations. (*b*) Path diagram depicting only significant effects from the full model (see electronic supplementary material, table S4). Line width indicates standardized parameter estimate and colour indicates negative (red) or positive (blue) directionality of effects. *p < 0.05, **p < 0.001, ***p < 0.0001.

**Figure 4.**
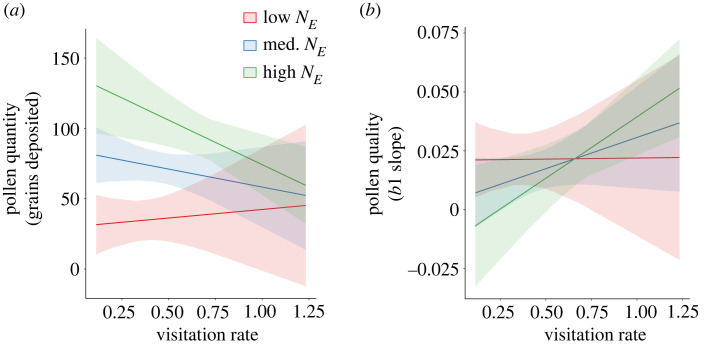
Effect plots depicting the interactions between the effective population size of plant populations and pollinator visitation rate on (*a*) quantity and (*b*) quality components of pollen limitation.

The full structural equation model is depicted in figure 3*a*, while significant effects are provided in figure 3*b*. Interactions between pollinator visitation and effective population size impacted both pollen quantity and quality limitation (figure 3*b*; electronic supplementary material, table S4). Pollen deposition increased with *N_E_* (figure 3*b*; *p* < 0.0001). Variation in pollinator visitation had little effect on pollen deposition in small populations but, contrary to expectations, increased visitation reduced pollen deposition in populations with high *N_E_* (figures 3*b* and 4*a*; visitation × effective population size *p* = 0.001). Pollinator visitation increased stigmatic pollen quality only in effectively larger populations (figures 3*b* and 4*b*; visitation × effective population size *p* = 0.002).

Pollen quantity and quality each impacted separate metrics of reproductive success (figure 3*b*). Increased pollen quantity resulted in increased fruit set (*p* = 0.01) but had no effect on seed set (*p* = 0.38). Conversely, increased pollen quality enhanced seed set (*p* < 0.0001; figure 3*b*) but had no effect on fruit set (*p* = 0.13). The overall model demonstrates interactive effects of pollinators and plant effective population size on plant reproduction through their effects on pollen quantity and quality limitation.

### (d) Pollen deposition as a characteristic of populations independent of the pollination environment

**Figure 5.**
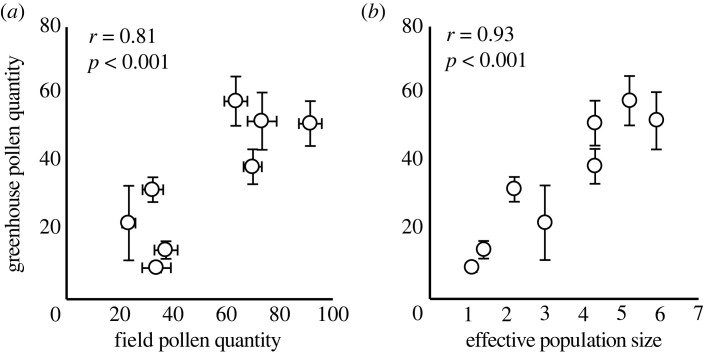
(*a*) The relationship between average population-level pollen quantity measured in the field, and pollen quantity measured from controlled crosses in a pollinator-free greenhouse. (*b*) The relationship between effective population and size pollen quantity measured from controlled crosses. Error bars depict 1 s.e.

To dissect whether populations with larger effective population size have higher pollen quantity irrespective of the pollination environment, we scored pollen loads deposited on stigmas from standardized hand pollinations in the greenhouse using a subset of populations. The average size of the stigmatic pollen load resulting from hand pollinations in a pollinator-free greenhouse was positively correlated with average population-level pollen deposition measured in the field (figure 5*a*; *r* = 0.81, *p* < 0.001). Likewise, pollen deposition in the greenhouse was strongly positively correlated with *N_E_* (figure 5*b*; *r* = 0.93, *p* < 0.001). Average anther number per flower was not correlated with effective population size, pollen deposition in the field, or pollen deposition in the greenhouse (all *r* < 0.31, all *p* > 0.50), suggesting that reduced pollen production is associated fewer pollen grains per anther rather than fewer anthers per flower. These results support that variation in pollen quality limitation in the field is driven, in large part, by reduced pollen production in genetically smaller populations rather than reduced pollinator visitation rates.

## 4. Discussion

Our results show that the genetic effective population size of plants can contribute strongly to variation in PL among populations, and that it modifies the impact of pollinators on PL. In plant species with the capacity for clonality, reduced investment in sexual reproduction is expected in populations that are highly clonal [24]. Our data support this prediction and show that divestment in pollen in effectively smaller populations of a clonal plant exacerbates pollen quantity limitation. Furthermore, results support that increased pollinator visitation rates alleviated pollen quality limitation in effectively larger plant populations, but pollinator visitation had very little effect on pollen quality limitation in effectively smaller populations. This result likely reflects low S-allele diversity in effectively smaller populations [27]. Finally, the quantity and quality components of PL affected separate female reproductive output metrics—fruit set was mediated by pollen quantity, while seed set was mediated by pollen quality. Together, our results suggest that efforts to support pollinators will be most beneficial for the reproduction of plants in genetically diverse populations.

### (a) Population genetic and pollination impacts on pollen quantity

Our prediction that effectively smaller populations would experience stronger pollen quantity limitation was supported by observational data in natural populations and further bolstered by controlled crosses in a pollinator-free environment. Effective population size had a direct positive effect on the quantity of pollen deposited on stigmas in natural populations. When we eliminated variation in pollinator visitation by conducting crosses in the greenhouse, stigmatic pollen deposition was also lower in populations with smaller effective population size. Variation in pollen deposition was not explained by variation in pistil number per flower across populations. Together, these results suggest that populations that are more clonal produce less pollen which contributes to reduced pollen deposition. This finding would not have been revealed without controlled crosses, because in the field pollinator visitation rates were positively correlated with effective population size. One potential explanation for the positive correlation between pollinator visitation and effective population size is that flowers in effectively larger populations provide a better pollen source than those in effectively smaller populations. Regardless of the positive correlation between effective population size and pollinator visitation, effective population size was the only factor that had a direct effect on pollen quantity limitation.

Contrary to expectations, increased pollinator visitation was associated with reduced pollen deposition in populations with higher effective population size. Because increased pollinator visitation results in higher rates of pollen export in *A. anserina* (e.g. [37]), we posit that increased visitation reduced the amount of within-flower autonomous pollen that was passively deposited on stigmas within flowers. Another potential explanation for the decrease in pollen deposition with increased pollinator visitation could be that higher rates of visitation deplete pollen if it is consumed or used to provision offspring [51]. Higher rates of floral visitation by small solitary bees have been linked with increase population-level PL in other systems [19]. While higher stigmatic pollen loads with higher pollinator visitation holds in many taxa [52,53] our results caution against using pollen deposition as a proxy for pollinator visitation in all systems.

### (b) Population genetic and pollination impacts on pollen quality

As predicted, increased pollinator visitation enhanced the quality of the stigmatic pollen load, but only in effectively large populations, driving an interaction between *N_E_* and pollinator visitation rate on pollen quantity. This result most likely reflects higher diversity of self-incompatibility alleles in effectively larger populations [27]. In effectively small populations, increased pollination services may fail to increase the quality of the stigmatic pollen load unless pollinators introduce novel S-alleles from other populations. Our results are in line with Young & Pickup [12] who showed that populations with larger census size had higher seed set because of higher estimated S-allele diversity but not because of increased pollen deposition. Conversely, one study found no association between PL and population genetic diversity or S-allele diversity in *Prunus virginiana*, though only three populations were sampled [54]. In the populations of *A. anserina* studied here, it will be important to use an ecological genetic crossing design and/or molecular approaches to determine the degree to which effective population size and S-allele diversity are positively correlated.

### (c) Links between pollen limitation and reproductive output

Metrics of pollen quantity and pollen quality limitation each affected a separate component of seed output. Specifically, populations with larger stigmatic pollen loads on average converted more flowers to fruit, while those with stigmatic loads of higher quality converted more ovules per flower to seed. Interestingly, fruit set and seed set were only modestly positively correlated (and not significantly so), and metrics of pollen quantity and pollen quality were completely uncorrelated. Using structural equation modelling, we were able to link genetic (effective population size) and ecological (pollinator visitation) factors to distinct metrics of reproductive output via their effects on the components of PL. Higher pollinator visitation rates resulted in higher seed set via increasing pollen quality in effectively larger populations. However, higher pollinator visitation rates had a negative effect on fruit set in effectively larger populations. Thus, results suggest that pollinators are most important for mediating plant reproductive output in effectively larger populations of SI species.

## 5. Conclusion

Plant population sizes are expected to be continually eroded by habitat fragmentation and climate change which should increase levels of inbreeding [16]. While increased pollen transfer among individuals should reduce inbreeding, thereby increasing genetic diversity, this is unlikely to be the case in small populations with self-incompatibility systems. Especially in SI organisms with the capacity for clonality, population sizes may become so small that sexuality is lost altogether. Our results documented the depression of sexual reproduction in effectively small plant populations regardless of the level of pollination services. However, when plant populations have sufficient genetic diversity, pollination services can enhance plant reproduction through the delivery of higher quality pollen.

## Data Availability

All data and code are archived in the Dryad Digital Repository: https://datadryad.org/stash/share/FV8p09jqcs8JcSCYzMk8V_CKVgG4B3BVWo7GenxnXD0 [55]. The data are provided in electronic supplementary material [56].
